# No interactions between heparin and atacicept, an antagonist of B cell survival cytokines

**DOI:** 10.1111/bph.14811

**Published:** 2019-10-15

**Authors:** Christine Kowalczyk‐Quintas, Daniela Willen, Laure Willen, Michaela Golob, Sonia Schuepbach‐Mallepell, Benjamin Peter, Mahya Eslami, Michele Vigolo, Hervé Broly, Eileen Samy, Özkan Yalkinoglu, Pascal Schneider

**Affiliations:** ^1^ Department of Biochemistry University of Lausanne Epalinges Switzerland; ^2^ Clinical Pharmacology, Quantitative Pharmacology, Global Early Development Merck KGaA Darmstadt Germany; ^3^ EMD Serono Research & Development Institute, Inc. Billerica MA USA; ^4^ Biotech Process Sciences Merck KGaA Corsier‐sur‐Vevey Switzerland

## Abstract

**Background and Purpose:**

The TNF family ligands, B cell activating factor of the TNF family (BAFF, also known as B lymphocyte stimulator, BLyS) and a proliferation‐inducing ligand (APRIL), share the transmembrane activator and calcium‐modulator and cyclophilin ligand (CAML)‐interactor (TACI) as one of their common receptors. Atacicept, a chimeric recombinant TACI/IgG1‐Fc fusion protein, inhibits both ligands. TACI and APRIL also bind to proteoglycans and to heparin that is structurally related to proteoglycans. It is unknown whether the portion of TACI contained in atacicept can bind directly to proteoglycans, or indirectly via APRIL, and whether this could interfere with the anti‐coagulant properties of heparin.

**Experimental Approach:**

Binding of atacicept and APRIL to proteoglycan‐positive cells was measured by FACS. Activities of heparin and atacicept were measured with activated factor Xa inhibition and cell‐based assays. Effects of heparin on circulating atacicept was monitored in mice.

**Key Results:**

Atacicept did not bind to proteoglycan‐positive cells, but when complexed to APRIL could do so indirectly via APRIL. Multimers of atacicept obtained after exposure to cysteine or BAFF 60‐mer bound directly to proteoglycans. Atacicept alone, or in complex with APRIL, or in a multimeric form did not interfere with heparin activity in vitro. Conversely, heparin did not influence inhibition of BAFF and APRIL by atacicept and did not change circulating levels of atacicept.

**Conclusions and Implications:**

Lack of detectable interference of APRIL‐bound or free atacicept on heparin activity makes it unlikely that atacicept at therapeutic doses will interfere with the function of heparin in vivo.

**Abbreviations**
APRIL: a proliferation‐inducing ligand
BAFF: B cell activating factor of the TNF family
BCMA: B cell maturation antigen
BLyS: B lymphocyte stimulator
TACI: transmembrane activator and calcium‐modulator and cyclophilin ligand (CAML)‐interactor

**What is already known**

- Interactions of the receptor TACI and its ligand APRIL with proteoglycans are inhibited by heparin.
- Atacicept contains a portion of TACI that may potentially interfere with heparin, directly or indirectly.

**What this study adds**

- Neither atacicept alone, nor atacicept–APRIL complexes, nor atacicept multimers interfere with heparin activity.
- Conversely, heparin does not interfere with atacicept activity and circulating levels of atacicept in mice.

**What is the clinical significance**

- There is no reason to suspect drug interference between atacicept and heparin or heparin‐like anticoagulants.

## 1 INTRODUCTION

The B cell activating factor of the TNF family (http://www.guidetopharmacology.org/GRAC/LigandDisplayForward?ligandId=5069, also known as B lymphocyte stimulator, BLyS) and a proliferation‐inducing ligand (http://www.guidetopharmacology.org/GRAC/LigandDisplayForward?ligandId=5068) play important roles in the generation, maintenance, and function of peripheral B cells at various stages of maturation (reviewed in (Mackay & Schneider, 2009). To exert these effects, BAFF binds three different receptors, the BAFF receptor (http://www.guidetopharmacology.org/GRAC/ObjectDisplayForward?objectId=1886), the transmembrane activator and calcium‐modulator and cyclophilin ligand (CAML)‐interactor (http://www.guidetopharmacology.org/GRAC/ObjectDisplayForward?objectId=1885), and the B cell maturation antigen (http://www.guidetopharmacology.org/GRAC/ObjectDisplayForward?objectId=1889), whereas APRIL only binds to TACI and BCMA (reviewed in Mackay & Schneider, 2009). Because of their importance in B cell survival, BAFF alone (Furie et al., 2011; Navarra et al., 2011) or BAFF and APRIL (Isenberg et al., 2014; Merrill et al., 2018) have been targeted in patients with autoimmune diseases involving pathogenic auto‐reactive B cells. The anti‐BAFF monoclonal antibody http://www.guidetopharmacology.org/GRAC/LigandDisplayForward?ligandId=6887 is approved by the Food and Drug Administration for the treatment of systemic lupus erythematosus (SLE; Furie et al., 2011; Navarra et al., 2011), while the dual BAFF and APRIL inhibitor http://www.guidetopharmacology.org/GRAC/LigandDisplayForward?ligandId=9517, consisting of the extracellular portion of TACI fused to the Fc portion of human IgG1 modified to reduce interactions with complement and antibody receptors, is in clinical development for the same disease (Isenberg et al., 2014; Merrill et al., 2018). When cleaved at furin consensus sites, the trans‐membrane proteins APRIL and BAFF are released as soluble cytokines (see Bossen & Schneider, 2006). APRIL is a basic, positively charged protein that can bind to negatively charged proteoglycans, in particular through an amino acid sequence located after the furin cleavage site (Hendriks et al., 2005; Ingold et al., 2005). This binding is believed to concentrate and cross‐link APRIL on cells to facilitate encounter with TACI and BCMA and possibly create and stabilize chemical gradients of APRIL (Huard et al., 2008; Kimberley et al., 2009). In B cells, TACI and heparan sulfate proteoglycans were both required for APRIL to induce IgA production (Sakurai et al., 2007). TACI was further reported to interact with proteoglycans, which under certain conditions was sufficient to activate TACI signalling (Bischof et al., 2006). In another study, TACI‐Fc bound syndecan‐1‐positive multiple myeloma cells unless http://www.guidetopharmacology.org/GRAC/LigandDisplayForward?ligandId=4214 was present or heparan sulfate chains were hydrolyzed by treatment with heparitinase (Moreaux et al., 2009). Interactions of APRIL and TACI with proteoglycans could invariably be disrupted by heparin in all studies where this has been tested.

In blood, the serine protease inhibitor anti‐thrombin limits activation of coagulation proteases such as factor X and thrombin. The activity of anti‐thrombin is strongly activated by heparin and its low MW forms, which are highly negatively charged sulfated glycosaminoglycans widely used as anti‐coagulants. Low MW heparins directly bind anti‐thrombin, inducing conformational changes that place the reactive centre loop in an ideal position to inhibit activated factor X, while longer forms of heparin can additionally create a physical bridge between anti‐thrombin and thrombin to reinforce inhibition (Johnson, Li, Adams, & Huntington, 2006; Li, Johnson, Esmon, & Huntington, 2004). Heparin has long been described to release into the blood circulation a “clearing factor” for lipids, identified as lipoprotein lipase (Korn, 1955). Lipoprotein lipase is a soluble enzyme that remains bound to the surface of endothelial cells by electrostatic interactions with heparan sulfate proteoglycans. It can be released from endothelial cells with heparin (Shimada, Gill, Silbert, Douglas, & Fanburg, 1981). TACI, APRIL, and anti‐thrombin all can bind to heparin. This raises the question of whether atacicept, which contains a portion of extracellular TACI, may sequester heparin directly or indirectly, via APRIL, and interfere with its anti‐coagulant action. Here, we show that this is unlikely to be the case.

## 2 METHODS

### 2.1 Human samples

Human serum samples were from patients with SLE who were enrolled in the randomized, double‐blind, APRIL‐SLE trial (http://ClinicalTrials.gov Identifier NCT00624338). In addition to standard‐of‐care therapy, patients received 150‐mg atacicept subcutaneously twice a week for 4 weeks and then weekly for 48 weeks (Isenberg et al., 2014). Other sera samples obtained at various time points after a single subcutaneous administration of 150‐mg atacicept in healthy subjects were from study EMR700461‐022. Trials were conducted according to the Declaration of Helsinki, applicable local regulations, and the International Council for Harmonization guideline for Good Clinical Practice.

### 2.2 Mice, cells, and reagents

Animal studies are reported in compliance with the ARRIVE guidelines (Kilkenny et al., 2010) and with the recommendations made by the *British Journal of Pharmacology*. Mouse and human APRIL both bind to proteoglycans in a heparin‐sensitive manner (Hendriks et al., 2005; Ingold et al., 2005). In addition, human TACI binds to mouse APRIL and BAFF (Bossen et al., 2006). For these reasons, mice are a relevant model to test interactions of atacicept with heparin, endogenous APRIL, and PGs. Mice were handled according to Swiss Federal Veterinary Office guidelines. Experiments with animals performed in this study were approved by the local institutional animal care and use committee and by the Office Vétérinaire Cantonal du Canton de Vaud (authorization 1370.6 to PS). Female C57BL/6 WT mice were purchased from Envigo (Horst, Netherlands). Four to five animals per cage were housed in a specific pathogen‐free facility at 21°C, 50 ± 10% humidity, with a 14‐hr:10‐hr light/night cycle. Mice were provided with water at pH 2.8 and Global Rodent XP18 food (Kliba Nafag). Cages were enriched with tunnel kraft, dome, sizzle ball, and beech log (Serlab).

HEK293 (ATCC Cat# CRL‐1573, RRID:CVCL‐0045) and HEK293T (ATCC Cat# CRL‐3216, RRID: CVCL‐0063) cells were obtained from late Jürg Tschopp (University of Lausanne). HEK293 and HEK293‐hBAFF full‐544 cells (Schneider et al., 1999) were cultured in DMEM:F12 (1:1 v/v) supplemented with 2% fetal calf serum. 293T cells were cultured in DMEM containing 10% fetal calf serum. CHO‐S cells were from Thermoscientific (Cat# A1155701, RRID: CVCL‐7183). Jurkat BCMA:Fas‐2309 cl13 and Jurkat JOM2‐BAFFR:Fas‐2308 cl21 cells have been reported previously and were cultured in RPMI supplemented with 10% fetal calf serum (Bossen et al., 2008; Nys et al., 2013). These cell lines were not authenticated as their identity does not impact on results interpretation. hTACI‐Fc (containing amino acid residues 2–159, or 1–118, or 2–118, or 2–70, or 67–118, 67–110, or 30–110 of hTACI), hBCMA‐Fc (containing amino acid residues 1–51 or 1–54), mBAFFR‐Fc, http://www.guidetopharmacology.org/GRAC/FamilyDisplayForward?familyId=334#2325‐Fc, TNFR2‐Fc, Fc‐BAFF, and Fc‐APRIL were produced in CHO‐S cells cultured in OptiMEM without serum supplementation (or chemically defined MS‐CHO‐PM209 medium, a Merck Serono proprietary formulation supplied by Merck Millipore, for TACI‐Fc 30–110, and for TACI‐Fc 2–70 that was produced in both media) and purified on Protein A Sepharose or, when indicated, on Protein G Sepharose, essentially as described (Schneider, 2000). Flag‐ACRP‐hAPRIL (Flag‐APRIL; produced in HEK293 cells) and Flag‐mBAFF (produced by transient transfection in 293T cells) were purified on anti‐Flag agarose, as described (Schneider, 2000). BAFF 60‐mer was produced as described (Bossen et al., 2008; Vigolo et al., 2018). Plasmids used in this study are described in Table 1. Atacicept (TACI (30–110)‐Fc) and anti‐hTACI mouse monoclonal antibodies ATA1 (250.14.1.1.4.3), ATA2 (251.15.1.1.1.5), and ATA3 (251.10.1.4.2.1) were provided by Merck (Darmstadt, Germany). TNFR2‐Fc (http://www.guidetopharmacology.org/GRAC/LigandDisplayForward?ligandId=6789, registered trade name Enbrel) was purchased from the Pharmacy of Lausanne University Hospital (CHUV). Anti‐mouse BAFF monoclonal antibodies 5A8 (rat IgG1) and Sandy2 (mouse IgG1) were as described (Kowalczyk‐Quintas et al., 2016). 5A8 (Enzo Life Sciences Cat# ALX‐804‐158‐C100, RRID:AB_2050785) and Sandy‐2 (Adipogen Cat# AG‐20B‐0063PF, RRID:AB_2490279) are also commercially available. Biotinylated anti‐Flag M2 antibody (Sigma‐Aldrich Cat# F9291, Lot #087K6004, RRID:AB_439689), fatty acid‐free bovine serum albumin, Zwittergent, and poly‐lysine were from Sigma‐Aldrich. The EnzCheck lipase substrate, a triglyceride analogue, was purchased from ThermoFischer. Heparin (Liquemin 5,000 IU·ml^−1^) was from DrossaPharm (Basel, Switzerland). Biophen heparin (LRT) and Biophen heparin calibrator kits were from Hyphen (Neuville‐sur‐Oise, France). AlphaLISA acceptor beads and streptavidin‐coupled AlphaLISA donor beads were purchased from PerkinElmer.

**Table 1:** Plasmids used in this study

Plasmid	Designation	Protein encoded	Vector
ps544	hBAFF	hBAFF (1–285)	PCR3
ps657	Flag‐mBAFF	HA signal‐Flag‐GPGQVQLHVD‐mBAFF (aa 127–309)	PCR3
ps882	hTACI (2–159)‐Fc	HA signal‐LD‐hTACI (aa 2–159)‐VD‐hIgG1 (aa 245–470)	PCR3
ps926	hTACI (2–118)‐Fc	HA signal‐LD‐hTACI (aa 2–110)‐VD‐hIgG1 (aa 245–470)	PCR3
ps927	hTACI (2–70)‐Fc	HA signal‐LD‐hTACI (aa 2–70)‐VD‐hIgG1 (aa 245–470)	PCR3
ps930	hEDAR‐Fc	hEDAR (aa 1–183)‐VD‐hIgG1 (aa 245–470)	PCR3
ps952	hTACI (67–118)‐Fc	HA signal‐LE‐hTACI (aa 67–118)‐VD‐hIgG1 (aa 245–470)	PCR3
ps1011	hBCMA (1–54)‐Fc	Ig signal‐VQC‐hBCMA (aa 1–54)‐VD‐PreScission‐hIgG1 (aa 245–470)	PCR3
ps1088	Flag‐ACRP‐hAPRIL	HA signal‐Flag‐GPGQVQLQ‐hACRP30 (aa 16–108)‐MQ‐hAPRIL (aa 105–250)	PCR3
ps1155	Fc‐hAPRIL A88	HA signal‐LD‐ hIgG1 (aa 245–470)‐RS‐CamLinker‐GSLQ‐hAPRIL (aa 105–250)	PCR3
ps1377	pMSCV‐puro	Modified pMSCV‐puro (Clonetech) with HindIII‐Bg1II‐EcoRI‐NotI‐XhoI‐HpaI‐ApaI cloning sites	ps1377
ps3139	hTNFR2‐PS‐Fc	hTNFR2 (aa 1–257)‐VDHHHHHHLD‐PreScision‐hIgG1 (aa 245–470)	PCR3
ps2297	mBAFFR‐Fc	HA signal‐LD‐mBAFFR (aa 2–70)‐VD‐hIgG1 (aa 245–470)	PCR3
ps2308	hBAFFR:Fas	HA signal‐LE‐hBAFFR (aa 2–71)‐EFGSVD‐hFas (aa 169–335)	ps1377
ps2309	hBCMA:Fas	Ig signal‐VQCEVKLVPRGS‐hBCMA (aa 2–54)‐VD‐hFas (aa 169–335)	ps1377
ps2565	His_6_‐hBAFF	MRGSHHHHHH‐h BAFF (aa 134–245)	pQE9
ps2825	Fc‐hBAFF	HA signal‐LD‐hIgG1 (aa 245–470)‐RS‐CamLinker‐GSLQ‐hBAFF (aa 136–285)	PCR3
ps3426	hTACI (1–118)‐Fc	Modified Ig signal hTACI (aa 1–118) hIgG1 (aa 245–470; L258E, A353S, P354S)	PCR3
ps3449	hBCMA (1–51)‐Fc	Modified Ig signal hBCMA (aa 1–51)‐VDHHHHHHLD‐PreScission‐hIgG1 (aa 245–470)	PCR3
ps3613	hTACI (67–110)‐Fc	Modified Ig signal hTACI (aa 67–110)‐VD‐PreScission‐hIgG1 (aa 245–470)	PCR3
ps3825	hTACI (31–110)‐Fc	Modified Ig signal hTACI (aa 31–110)‐VD‐hIgG1 (aa 245–470)	

*Note*. HA signal = MAIIYLIILLFTAVRG. Ig signal = MNFGFSLIFLVLVLKG. CamLinker = PQPQPKPQPKPEPEGS. Modified Ig signal = METDTLLLWVLLWVPGVHG. PreScission = LEVLFQGP. Flag = DYKDDDDK.

#### 2.2.1 Biotinylation

Antibodies in 1 ml of 0.1‐M Na‐borate pH 8.8 were biotinylated with 100‐μg EZ‐Link‐Sulfo‐NHS‐LC‐biotin (Pierce) per milligram of antibody for 2 hr at room temperature. The reaction was stopped by the addition of 10 μl of 1‐M NH_4_Cl, and buffer was then exchanged for PBS in a 30‐kDa cut‐off centrifugal device (Millipore).

#### 2.2.2 ELISA

elisa plates were coated with atacicept at 1 μg·ml^−1^ in PBS, blocked, and incubated with the indicated concentrations of Flag‐APRIL. Binding of Flag‐APRIL was revealed with appropriate anti‐Flag secondary reagents.

#### 2.2.3 SDS‐PAGE and western blot

The immuno‐related procedures used comply with the recommendations made by the *British Journal of Pharmacology*. SDS‐PAGE was performed under reducing or non‐reducing conditions, as mentioned. Coomassie blue staining was performed with a semidry iD Stain System (Eurogentech). Western blots were performed on nitrocellulose membranes according to standard procedures and reveal with horseradish peroxidase‐coupled donkey anti‐human IgG (Jackson ImmunoResearch Labs #709‐036‐149, RRID:AB_2340498; 1/5,000), or with anti‐TACI ATA1 antibody at 1 μg·ml^−1^, followed by HRP‐coupled goat anti‐mouse IgG (Jackson ImmunoResearch Labs Cat #115‐035‐146, RRID:AB_2307392; 1/5,000). Blots were revealed with ECL.

#### 2.2.4 Preparation of atacicept multimers

Eighty microlitres of atacicept at 75 mg·ml^−1^ was mixed with 80 μl of 20‐mM Na‐phosphate pH 7, 10 mM EDTA, with or without 20 mM of freshly prepared cysteine. The mixture was incubated for 48 hr at 37°C with agitation and then size‐fractionated by gel permeation chromatography.

#### 2.2.5 Preparation of Fc fragment

Four hundred microlitres of TNFR2‐His_6_‐Fc at 5 mg·ml^−1^ in PBS, with a PreScission cleavage site between the His tag and Fc, was digested for 48 hr at 4°C with 40 U·ml^−1^ of GST‐PreScission protease (GE Healthcare). Fc was recovered by affinity purification on Protein A‐Sepharose, followed by gel permeation chromatography on Superdex‐200 column and concentration on a 30‐kDa cut‐off centrifugal device.

#### 2.2.6 Gel permeation chromatography

Protein A or Protein G‐purified TACI‐Fc or BCMA‐Fc were loaded in a volume of 400 μl on a Superdex‐200 Increase column (GE Healthcare) eluted in 20‐mM HEPES pH 8.2, 130‐mM NaCl with online absorbance monitoring at 280 nm, and 1‐mL fraction collection. Fractions of interest were quantified by measuring absorbance at 280 nm and by using the theoretical extinction coefficient. If necessary, they were first concentrated on 30‐kDa cut‐off centrifugal devices. For the preparation of atacicept multimers, samples were loaded in a volume of 160 μl on a Superdex‐200 Increase column eluted in 0.1‐M ammonium acetate pH 7.

#### 2.2.7 FACS

HEK293 or 293T cells were stained for 20 min on ice with Flag‐APRIL or Fc‐containing proteins at the indicated concentrations. Atacicept‐containing sera were used at a dilution of 1/5, followed by five washes, for which care was taken to carefully suspend cells after each wash and to drain all of the wash liquid to bring levels of soluble serum human IgGs below the level that could quench the secondary staining reagent. Binding of Flag‐APRIL was revealed with 2 μg·ml^−1^ of biotinylated anti‐Flag antibody followed by phycoerythrin‐coupled streptavidin (1/500; eBiosciences, Cat #12‐4317‐87, Lot #E01657‐1636), while binding of Fc‐containing proteins was revealed with 1 μg·ml^−1^ of phycoerythrin‐coupled goat anti‐human IgG (1/500; SouthernBiotech Cat #2040‐09, Lot #01316‐Q487, RRID:AB_2795648). When present, heparin at a final concentration of 8 IU·ml^−1^ was added together with recombinant proteins, unless stated otherwise. Samples were analysed with an Accuri 6 flow cytometer (BectonDickinson).

#### 2.2.8 Cytotoxicity assay

Cytotoxicity assays using BAFFR:Fas or BCMA:Fas cells were performed essentially as described (Schneider, Willen, & Smulski, 2014). Briefly, tests were performed with 3 to 4 × 10^4^ cells per well in flat‐bottomed 96‐well plates in a final volume of 100 μl of RPMI 10% fetal calf serum and in the presence of the indicated concentrations of agonists (Fc‐BAFF or Fc‐APRIL), inhibitors (atacicept or atacicept in mouse plasma at a final plasma dilution of 1/1,000), and heparin. The measurement of atacicept activity in plasma was performed independently either in the presence or in the absence of heparin at 8 IU·ml^−1^. After an overnight incubation at 37°C, 5% CO_2_, cell viability was monitored with a colorimetric (PMS/MTS) test. When active atacicept was measured in plasma of mice treated with heparin, a correction factor was applied to account for blood dilution induced by the intravenous injection, assuming a blood volume of 80 ml·kg^−1^.

#### 2.2.9 Activated factor X inhibition assay

Ten microlitres of calibration plasma (0, 0.38, 0.74, 1.12, or 1.55 IU·ml^−1^ heparin), 10 μl of potential inhibitors (PBS, 90 μg·ml^−1^ poly‐lysine in PBS, 100 μg·ml^−1^ recombinant proteins in PBS, undiluted human sera), 50 μl of chromogenic factor X substrate, and 50 μl of bovine factor Xa solution were mixed in a 96‐well plate and incubated for 1 min at 37°C. The reaction was terminated by the addition of 50 μl of 50‐mM Na‐citrate pH 2.7, and absorbance was read at 405 nm. When two proteins were mixed, each was at a concentration of 100 μg·ml^−1^. To correct for intrinsic coloration of sera, background absorbance determined from reactions in which Na‐citrate pH 2.7 was added before bovine factor Xa was subtracted. For the determination of heparin activity in mouse plasma, the same test was done, using 10 μl of calibration plasma without heparin mixed with 10 μl of plasma diluted 1/25 in PBS.

#### 2.2.10 Atacicept and heparin administration in mice

Eight‐week‐old female mice were acclimated for a week upon arrival, attributed randomly to experimental groups and housed so that mice from a given time point were distributed in different cages. Power calculation: group size of six animals per group was chosen to detect differences of one third between conditions, assuming variation coefficients of 20%. Mice weighing 17.4 g on average (range 16.2 to 18.5 g) received atacicept subcutaneously at 5 mg·kg^−1^ on Day 0 or were left untreated. One, 3, 7, or 14 days after atacicept administration, blood (~200 μl) was collected from the facial vein, located just beneath the skin immediately caudal to the facial vibrissae at the corner of the jaw, with 5‐mm Goldenrod animal lancets (Braintree Scientific) under anaesthesia with isoflurane. Blood was collected in EDTA‐containing tubes (to get 5‐mM EDTA final) for plasma preparation. Within 30 min after blood withdrawal, mice received heparin 200 IU·ml i.v. at 1 IU·g^−1^, were left for 10 min, and then killed by CO_2_ inhalation. Plasma was prepared on EDTA from blood collected by cardiac puncture (at about 20 min post administration of heparin). For cardiac puncture, the animal is placed on the back, and a 0.5 × 16 mm 25G ^5^/_8″_ needle is inserted slightly left of and under the sternum, directly towards the head the animal, at an angle of 20–30° from horizontal. For the Day 14 time point, when atacicept plasma levels were anticipated to be closer to background, 10 mice were used instead of six. This does not introduce heterogeneity in group size as statistical analysis was performed between conditions at a given time point, and not between time points. Experiments were not performed blinded (all mice received the same dose of atacicept on Day 0, then heparin after different days, and plasma collection methods before and after heparin administration were distinct), but statistical tests for in vivo experiments were performed blinded by an independent person.

#### 2.2.11 Lipase assay

The assay was performed essentially as described (Basu, Manjur, & Jin, 2011). Briefly, 1 μl of plasma was incubated in black 96‐well plates for 1 hr at 37°C in 100 μl of 150‐mM NaCl, 20‐mM Tris–HCl pH 8, 1.5% fatty acid‐free BSA, 0.0125% Zwittergent, 0.1 U·ml^−1^ heparin, and 0.62‐μM EnzCheck lipase substrate. Fluorescence (excitation at 482 nm, read at 515 nm) was monitored every 2 min with a SpectraMax i3 device (Molecular devices). Lipase activity was expressed as fluorescence at 18 min – fluorescence at 8 min.

#### 2.2.12 AlphaLISA

One milligram of AlphaLISA acceptor beads (Perkin‐Elmer) was coupled for 24 hr at 37°C to 100 μg of monoclonal antibody ATA3 in 400 μl of 27‐mM Na‐phosphate pH 8, 5‐mM NaBH_3_CN, and 0.03% Tween‐20. The reaction was stopped with 100 μl of carboxymethoxylamine at 65 mg·ml^−1^ in 0.8‐M NaOH for 1 hr at 37°C. Beads were washed in 0.1‐M Tris–HCl pH 8 and then stored at 5 mg·ml^−1^ in PBS 0.05% Proclin‐300. Assays were performed in white shallow 384‐well plates by mixing 2 μl of sample (plasma diluted 1/100 in assay buffer) with 8 μl of 75 ng·ml^−1^ of biotinylated ATA2 and 0.2 μg of ATA3 acceptor beads in assay buffer. After 1‐hr incubation at room temperature, 0.4 μg of streptavidin‐coupled donor beads in 10 μl of assay buffer was added. Emission at 615 nm after excitation at 680 nm was recorded 10 to 15 min later with an Enspire plate reader (Perkin‐Elmer). For the acid‐dissociation procedure, assays were performed with 1.8‐μl sample (plasma diluted 1/100 in assay buffer), 0.2 μl of 4.25% *o‐*phosphoric acid, 0.8 μl of 350‐mM Tris HCl pH 10, 7.2 μl of a mix of 83 ng·ml^−1^ of biotinylated ATA2, and 0.2 μg of ATA3 acceptor beads. Samples were either incubated in acid for 20 min at room temperature, then neutralized with the mix of biotinylated ATA2, ATA3 beads and Tris, or incubated in pre‐neutralized acid mixed with biotinylated ATA2 and ATA3 beads. Samples were then incubated for 1 hr at room temperature and measured as described above. When atacicept was measured in plasma of mice treated with heparin, a correction factor was applied to account for blood dilution induced by the intravenous injection, assuming a blood volume of 80 ml·kg^−1^.

#### 2.2.13 Data and statistical analyses

The data and statistical analysis comply with the recommendations of the *British Journal of Pharmacology* on experimental design and analysis in pharmacology (Curtis et al., 2018). Normal distribution of data was confirmed with normality tests (D'Agostino Pearson normality test for *n* ≥ 8; Shapiro–Wilk test for *n* = 5 to 7). Comparison of two groups was performed with paired Student's *t* test. Comparison of multiple groups was performed by one‐way ANOVA followed, if *F* achieved *P* < .05 and Bartlett's test indicated no significant variance inhomogeneity, by Bonferroni's multiple comparison tests using GraphPad Prism, RRID:SCR_002798). Differences were considered statistically significant when *P* < .05.

#### 2.2.14 Nomenclature of targets and ligands

Key protein targets and ligands in this article are hyperlinked to corresponding entries in http://www.guidetopharmacology.org, the common portal for data from the IUPHAR/BPS Guide to PHARMACOLOGY (Harding et al., 2018), and are permanently archived in the Concise Guide to PHARMACOLOGY 2017/18 (Alexander et al., 2017).

## 3 RESULTS

### 3.1 Atacicept binds to proteoglycans indirectly via APRIL

**Figure 1:**
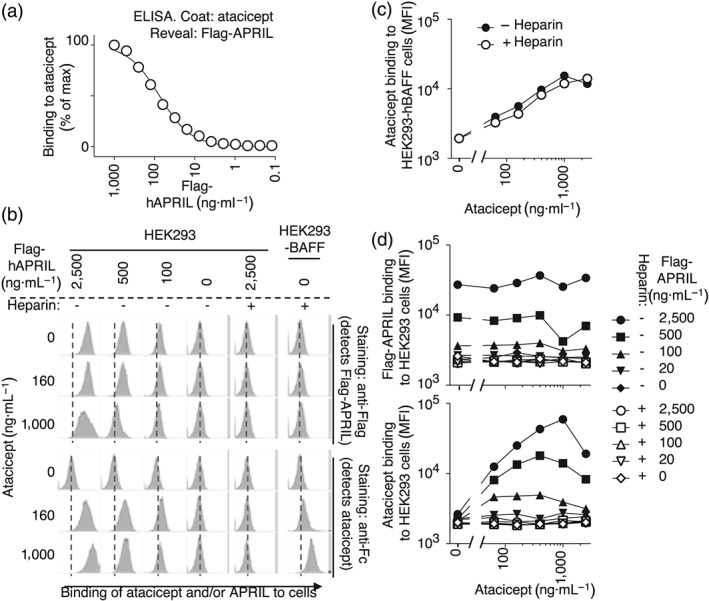
Atacicept does not bind proteoglycans directly but can do so via APRIL. (a) elisa assay to monitor the binding of Flag‐APRIL to coated atacicept. The experiment was performed three times. (b–d) HEK293 cells and the same cells stably transfected with full‐length human BAFF (HEK293‐BAFF) were stained with atacicept and/or Flag‐APRIL at the indicated concentrations, in the presence or absence of heparin (8 IU·ml^−1^). Binding of Flag‐APRIL and atacicept were respectively revealed with anti‐Flag and anti‐Fc secondary reagents. Results shown are from one of three similar experiments with similar results. (b) Selected histograms of the experiment described above in which the X‐axis displays fluorescence intensity on a log scale (3 × 10^2^ to 2 × 10^5^). (c) Mean fluorescence intensity (MFI) of atacicept binding to HEK293‐BAFF cells, in the presence or absence of heparin. (d) MFI of Flag‐APRIL or atacicept binding to HEK293 cells in the presence or absence. APRIL, a proliferation‐inducing ligand; BAFF, B cell activating factor of the TNF family

When coated to an elisa plate, the TACI‐Fc fusion protein atacicept bound a recombinant form of human Flag‐APRIL in a concentration‐dependent manner (Figure 1a). This recombinant Flag‐APRIL started at amino acid residue alanine 105 of APRIL, just after the furin cleavage site, and comprised the N‐terminal section of mature soluble APRIL that contributes to interaction with proteoglycans (Hendriks et al., 2005; Ingold et al., 2005). Flag‐APRIL stained HEK293 cells in a concentration‐dependent manner, and its binding was abolished by the addition of heparin (Figure 1b, top row). Flag‐APRIL binding still occurred in the presence of increasing concentrations of atacicept (Figure 1b, top part, and Figure 1d, top panel), indicating that although atacicept can bind to APRIL (Figure 1a), atacicept does not prevent the binding of APRIL to cell surface proteoglycans (Figure 1b,d). Atacicept could bind HEK293 cells stably transfected with full length BAFF, regardless of the presence or absence of heparin (Figure 1b, last column, and Figure 1c), but atacicept alone had no detectable interaction with untransfected HEK293 cells that express cell surface proteoglycans (Figure 1b, column 4). In the presence of APRIL, atacicept binding to HEK293 cells could be monitored by direct revelation of atacicept with anti‐Fc secondary reagents (Figure 1b, bottom two rows, and Figure 1d, bottom panel). This binding was inhibited in the presence of heparin (Figure 1b, column 5, and Figure 1d). Taken together, these data show that atacicept, unlike Flag‐APRIL, does not bind directly to HEK293 cells but can do so in the presence of Flag‐APRIL, in a heparin‐sensitive way. This suggests that atacicept does not bind cell surface proteoglycans directly but indirectly via binding to APRIL containing a proteoglycan interaction site.

### 3.2 Purified, dimeric forms of TACI‐Fc do not interact with cell surface proteoglycans

**Figure 2:**
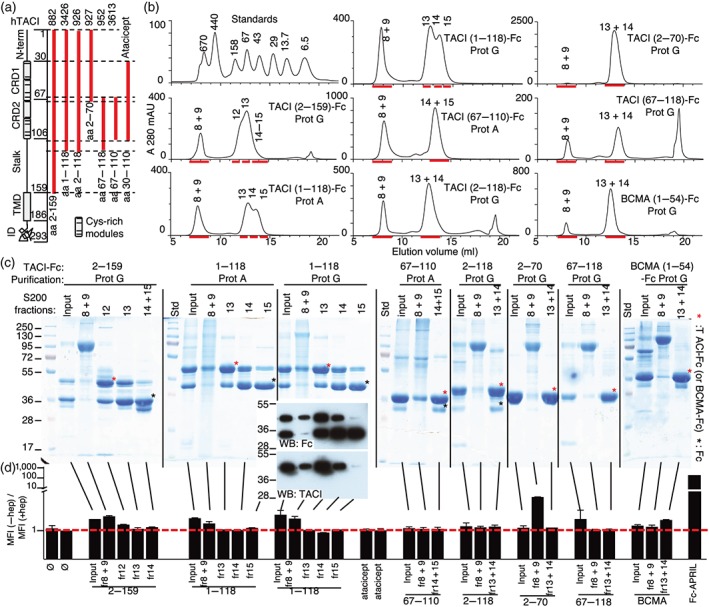
Dimeric recombinant TACI‐Fc does not bind to proteoglycans. (a) Schematic representation of human TACI. Portions of the extracellular domain of TACI contained in the different TACI‐Fc constructs are shown with thick vertical red lines. Numbers at the top refer to plasmid number encoding these proteins (see Table 1). aa, amino acid residues; CRD, cysteine‐rich domain; ID, intracellular domain; TMD, transmembrane domain. (b) UV elution profile of a size exclusion chromatography column loaded with a mixture of standards of indicated MW (in kDa) or with protein A or protein G‐purified TACI‐Fc or BCMA‐Fc constructs, as indicated. Fractions chosen for further analysis are indicated and underlined in red. (c) SDS‐PAGE (10% or 12% acrylamide) and Coomassie blue analysis of 10‐μg protein per lane of size‐fractionated proteins from panel (b). Input: Protein after Protein A or G affinity purification. MW standards indicated on the left are valid for the first gel. For the next ones, refer to standards (Std) shown on the left of each gel, knowing that red proteins correspond to 72 and 28 kDa, respectively. Target proteins are indicated with red asterisks, and presumptive Fc fragments with black asterisks. The insert shows a western blot analysis of 50 ng of the indicated fractions, revealed with anti‐human or anti‐TACI antibodies. (d) HEK293 cells were stained with the indicated proteins at 10 μg·ml^−1^, in the presence or absence of heparin. Results are expressed as the ratio of mean fluorescence intensity (MFI) in the absence of heparin to that in the presence of heparin. In the presence of heparin, all samples gave background MFI. The experiment was performed three times with similar results. BCMA, B cell maturation antigen; TACI, transmembrane activator and calcium‐modulator and cyclophilin ligand (CAML)‐interactor

The extracellular domain of TACI comprises 165 amino acids and contains two cysteine‐rich domains (CRD1 at position 33–67 and CRD2 at position 70–104) that mediate interactions with BAFF and APRIL. Atacicept, which contains amino acid residues 30–110 of TACI (TACI (30–110)‐Fc), does not bind to HEK293 cells (Figure 1), yet TACI is reported to bind to proteoglycans (Bischof et al., 2006; Moreaux et al., 2009). In an attempt to map the proteoglycan binding site of TACI, TACI‐Fc proteins containing various portions of extracellular TACI were studied (Figure 2a). Proteins obtained by affinity purification on Protein A or G were subsequently resolved by size exclusion chromatography. These preparations often contained high MW material that eluted in the void of the column (fraction 8 + 9) and a protein smaller in size that eluted in the inclusion volume of the column at sizes compatible with TACI‐Fc dimers (Figure 2b). In some instances, the included peak was split into a larger peak containing the TACI‐Fc fusion protein and a smaller peak containing a presumptive protease cleavage fragment with the Fc portion only (Figure 2b,c). In most cases, TACI‐Fc after purification on Protein A or G contained a set of contaminants migrating as a smear by SDS‐PAGE and that were mainly recovered in the void volume of the size exclusion column (Figure 2c). Several TACI‐Fc constructs post purification on Protein A or G bound weakly, compared to the positive control Fc‐APRIL, but specifically to HEK293 cells (Figure 2d). The high MW fractions, which contain some of the target proteins but also contaminants, often showed some binding to HEK293 cells, but purified, dimeric TACI‐Fc, including atacicept, did not bind to cell surface proteoglycans, regardless of the portion of TACI attached to the Fc (Figure 2d). Thus, we confirmed that atacicept does not bind to HEK293 cells and further extended this observation to several other dimeric TACI‐Fc constructs, including those containing the entire extracellular domain of TACI.

### 3.3 Atacicept and an atacicept‐APRIL complex do not interfere with the function of low MW heparin

**Figure 3:**
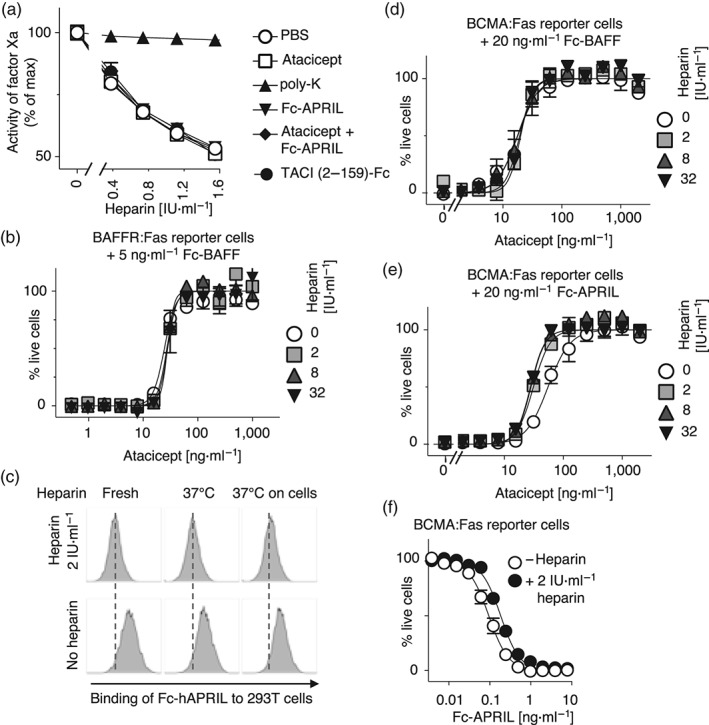
Atacicept does not interfere with heparin activity. (a) Heparin activity was measured as its ability to inhibit activated factor X (factor Xa) in the presence of human plasma (containing endogenous anti‐thrombin). The dose‐dependent inhibition of factor Xa by heparin was measured in the presence of the indicated proteins (each at 100 μg·ml^−1^), or of poly‐lysine (poly‐K; at 90 μg·ml^−1^). Mean ± *SEM* of duplicate measures. One representative experiment out of three similar ones with identical results is shown. (b) Reporter cells expressing the BAFFR:Fas fusion receptor were incubated overnight in the presence of a lethal (5 ng·ml^−1^) dose of Fc‐BAFF, in the presence of increasing concentrations of atacicept, and in the presence of the indicated amounts of heparin. Cell viability was measured with a colorimetric test. Mean ± *SEM* of duplicates. One experiment out of three is shown. (c) Heparin was incubated overnight at 37°C in medium, or with reporter cells in medium, after which time its ability to inhibit binding of Fc‐APRIL to HEK293 cells was monitored. The X‐axis displays fluorescence on a log scale (3 × 10^2^ to 5 × 10^4^). The experiment was performed three times. (d) Same as panel (b), but using BCMA:Fas reporter cells and a lethal (20 ng·ml^−1^) dose of Fc‐BAFF. Mean ± *SEM* of duplicates. The experiment was performed three times. (e) Same as panel (d), but with a lethal (20 ng·ml^−1^) dose of Fc‐APRIL. The experiment was performed three times with identical results. (f) BCMA:Fas reporter cells were cultured overnight with titrated amounts of Fc‐APRIL, plus or minus heparin. The experiment was performed three times with identical results. APRIL, a proliferation‐inducing ligand; BAFF, B cell activating factor of the TNF family; BAFFR, BAFF receptor; BCMA, B cell maturation antigen

When increasing concentrations of heparin were added to human plasma samples supplemented with both activated bovine factor X (factor Xa) and a chromogenic substrate for factor Xa, a dose‐dependent inhibition of factor Xa was observed that reflects inhibition of the protease in a ternary complex of anti‐thrombin (present in plasma), heparin, and factor Xa (Figure 3a). Not surprisingly, the action of poly‐anionic heparin was inhibited by poly‐cationic poly‐lysine (Figure 3a) but heparin activity was insensitive to the presence of atacicept (Figure 3a). Moreover, heparin activity was insensitive to the presence of a proteoglycan binding‐competent form of Fc‐hAPRIL plus or minus atacicept, and to (non‐size‐fractionated) TACI (2–159)‐Fc that, unlike atacicept, showed some binding to cell surface proteoglycans (Figure 2c,d). We conclude from these experiments that none of the APRIL or TACI‐containing reagents tested interfere with heparin activity, whether or not they bind to proteoglycans. In particular, atacicept, alone or in complex with APRIL, did not interfere with heparin activity in this assay.

### 3.4 Heparin does not interfere with the ability of atacicept to inhibit BAFF and APRIL

Atacicept does not bind to proteoglycans and does not inhibit heparin activity (Figures 1, 2, and 3a). Therefore, binding and inhibition of BAFF by atacicept is predicted to be insensitive to the presence of heparin. BAFF activity can be conveniently monitored using reporter cell‐based assays in which a Jurkat T cell line stably expressing fusion proteins containing the extracellular domains of BAFFR or BCMA, fused to the transmembrane and intracellular domains of the death receptor http://www.guidetopharmacology.org/GRAC/ObjectDisplayForward?objectId=1875, undergo Fas‐mediated death in response to BAFF (for BAFFR:Fas and BCMA:Fas reporter cells) or APRIL (for BCMA:Fas reporter cells; Schuepbach‐Mallepell et al., 2015). When reporter cells were exposed to a fixed, lethal dose of Fc‐BAFF, all cells died, unless increasing concentrations of atacicept were present to prevent death. The presence of up to 32 IU·ml^−1^ of heparin did not affect inhibition of Fc‐BAFF by atacicept (Figure 3b,d). It is noteworthy that heparin remained active after an overnight incubation on reporter cells, as measured by its ability to inhibit the binding of Fc‐APRIL to HEK293 cells (Figure 3c). In contrast to the inhibition of Fc‐BAFF, the inhibition of Fc‐APRIL was about twofold more efficient in the presence of heparin (Figure 3e), a result that can be readily explained by the twofold decrease of Fc‐APRIL activity on reporter cells in the presence of heparin (Figure 3f). Taken together, these results indicate that the activity of atacicept in blocking Fc‐BAFF and Fc‐APRIL is not altered in the presence of heparin but that the activity of Fc‐APRIL is slightly reduced in the presence of heparin.

### 3.5 Multimers of atacicept bind to cell surface proteoglycans

**Figure 4:**
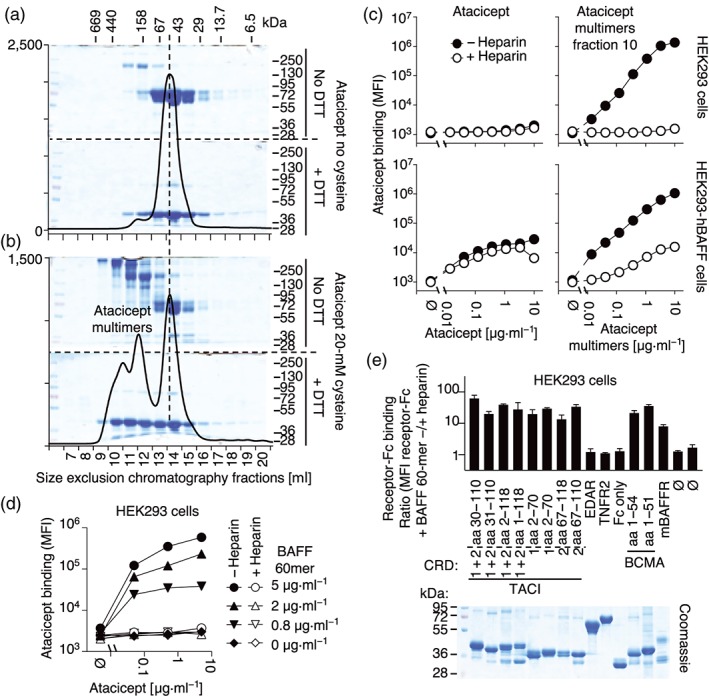
Multimers of atacicept bind to proteoglycans. (a) Size exclusion chromatography elution profile of atacicept incubated for 48 hr at 37°C in phosphate buffer without cysteine. Online monitoring of absorbance at 280 nm is shown as a line. Fractions analysed by SDS‐PAGE and Coomassie blue staining (20 μl) under non‐reducing (top gel) or reducing conditions (bottom gel) are shown in the background. Sizes of MW standards for SDS‐PAGE are shown on the right, and those for the size exclusion chromatography are shown on the top. The vertical dotted line marks the elution position of dimeric atacicept. (b) Same as panel (a), except that 20‐mM cysteine was included in the 48 hr incubation. The experiment was performed five times (but the Coomassie under non‐reducing conditions was performed twice). (c) HEK293 cells, with or without expression of membrane‐bound BAFF, were stained by FACS with atacicept (dimers) or atacicept multimers (from fraction 10, see panel (b)) at the indicated concentrations, in the presence or absence of heparin. MFI, mean fluorescence intensity. The experiment was performed three times. Points are monoplicates. (d) HEK 293 cells were incubated with the indicated concentrations of atacicept and BAFF 60‐mer, in the presence or absence of heparin, and stained by FACS anti‐hFc. The experiment was performed three times. (e) HEK293 cells were incubated with various Fc‐containing proteins (or no Fc‐containing protein: Ø) at 5 μg·ml^−1^ and with BAFF 60‐mer at 2 μg·ml^−1^ and then analysed by FACS anti‐hIgG. The ratio of the mean fluorescence intensity (MFI) of staining performed in triplicate without or with heparin are shown. Mean ± *SEM*. The experiment was performed three times with similar results. Ten micrograms of the Fc‐containing proteins (6 μg for mBAFFR‐Fc) used in these experiments were analysed by SDS‐PAGE and Coomassie blue staining under reducing conditions. BAFF, B cell activating factor of the TNF family; BAFFR, BAFF receptor

Although TACI‐Fc was previously shown to bind to proteoglycans (Bischof et al., 2006; Moreaux et al., 2009), our results with various dimeric TACI‐Fc constructs show no such interactions, suggesting that TACI‐Fc may require multimerization and/or aggregation to stably bind to proteoglycans. We found that incubation of atacicept with cysteine resulted in efficient formation of high MW, disulfide‐linked atacicept species, as judged by SDS‐PAGE analysis of fractions under reducing and non‐reducing condition (Figure 4a,b), which were indeed potent at binding cell‐surface proteoglycans in HEK293 cells (Figure 4c). In addition to proteoglycans, high MW atacicept species were also able to bind BAFF at the cell surface, but less efficiently than dimeric TACI‐Fc (Figure 4c). Twenty BAFF 3‐mer can assemble as a capsid‐like particle called BAFF 60‐mer, which does not dissociate upon atacicept binding (Vigolo et al., 2018). BAFF 60‐mer–atacicept complexes also bound cell‐surface proteoglycans in HEK293 cells, implying that oligomerization rather than disulfide‐bond formation is relevant for multimeric atacicept to bind to PG (Figure 4d). As cysteine did not induce multimerization of TACI‐CRD1‐Fc or Fc only, we used the BAFF 60‐mer‐mediated multimerization assay to attempt and map the proteoglycan‐binding site(s) of TACI. Control proteins (TNFR2‐Fc, EDAR‐Fc, or Fc alone) did not bind to HEK293 cells, even in the presence of BAFF 60‐mer. In contrast, in the presence of BAFF 60‐mer, all fusion proteins with a BAFF binding site (atacicept, TACI‐Fc constructs with CRD1, CRD2 or both, BCMA and BAFFR) bound HEK293 cells in a heparin‐inhibitable manner (Figure 4e). This suggests that weak proteoglycan‐binding units located in TACI CRD1, TACI CRD2, BCMA, BAFFR, and/or the Fc portion are reinforced upon oligomerization.

### 3.6 Atacicept in the human circulation does not bind to proteoglycans

**Figure 5:**
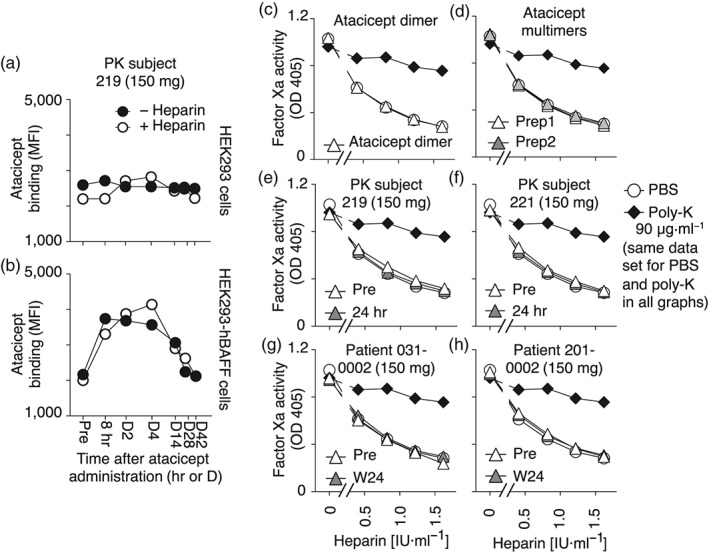
Atacicept in serum and atacicept multimers do not interfere with heparin activity. (a) HEK293 cells were stained by FACS, in the presence or absence of heparin, with atacicept contained in sera samples collected at the indicated time points from a healthy subject who received 150 mg atacicept. D, day; PK, pharmacokinetics. The experiment was performed twice with sera of subject 219, and once with sera of subject 221, treated identically, with similar results. (b) Same as panel (a), except that HEK293‐hBAFF cells were stained. (c) Heparin activity assay (inhibition of activated factor X) performed in the presence of PBS, 90 μg·ml^−1^ of poly‐lysine (Poly‐K), or 100 μg·ml^−1^ of dimeric atacicept. (d) Same as panel (c), but with two independent preparations of atacicept multimers at 100 μg·ml^−1^. (e, f) Same as panel (c), but with sera from healthy subjects, before or 24 hr after administration of 150 mg of atacicept. (g, h) Same as panels (e) and (f), but with sera of patients before or after 24 weeks of treatment with weekly administration of 150‐mg atacicept. Experiments in panels (c)–(h) were performed twice with similar results. Mean ± *SEM* of duplicates (*SEM* is smaller than symbol size). BAFF, B cell activating factor of the TNF family

After a single subcutaneous administration of 150‐mg atacicept in a healthy subject, atacicept in the circulation could be detected through its binding to BAFF‐expressing cells, but not to control cells, for up to 2 weeks after administration. Heparin did not affect staining, indicating that atacicept does not form multimers under these conditions, or that if they form, they are not retained into the serum fraction (Figure 5a,b).

### 3.7 Atacicept multimers and atacicept in the human circulation do not interfere with heparin activity

Two independent preparations of high MW atacicept obtained by exposure to cysteine did not interfere with heparin in the factor Xa inhibition assay (Figure 5c,d) despite marked binding to proteoglycans (Figure 4c). Sera of healthy subjects before or 24 hr after administration of 150‐mg atacicept (Figure 5e,f) and sera of patients before or after weekly treatments with high dose (150 mg) atacicept during 24 weeks had no inhibitory effect on clinically relevant amounts of heparin (Figure 5g,h). These results indicate that atacicept, even after prolonged contact with plasma or after forced multimerization, cannot interfere with heparin activity.

### 3.8 No evidence of atacicept interaction with proteoglycans in vivo

**Figure 6:**
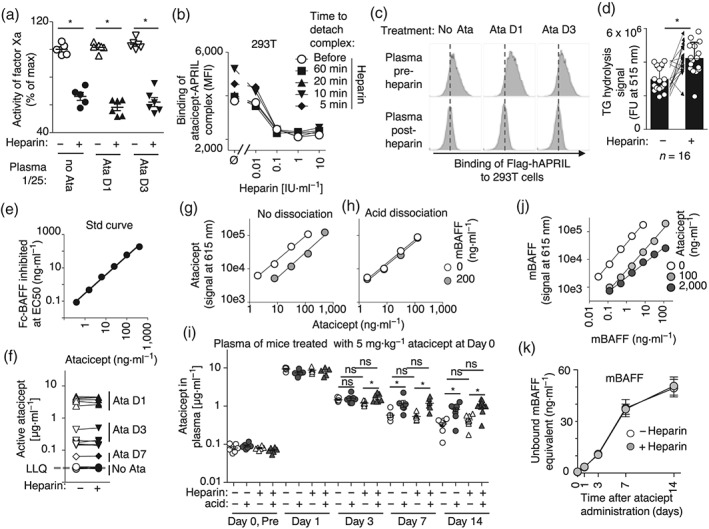
No evidence that atacicept binds to proteoglycans in vivo. Wild‐type mice were treated subcutaneously without (*n* = 5) or with atacicept at 5 mg·kg^−1^; 24 hr (D1, *n* = 6) or 72 hr (D3, *n* = 6) later, plasma was collected, and then 1 IU·g^−1^ of heparin was administered intravenously, and 20 min later, plasma was collected again. (a) Heparin activity in plasma was measured with the activated factor X (factor Xa) inhibition assay. *P<.05, significantly different as indicated; one‐way ANOVA with Bonferroni's multiple comparison test. (b) 293T cells stained with a complex of atacicept bound to Flag‐APRIL were exposed for the indicated times to the indicated concentrations of heparin, after which time cells were washed and bound atacicept was detected. The experiment was performed three times. (c) The activity of heparin in plasma was measured as its ability to prevent binding of Flag‐APRIL to 293T cells. In this assay, cells were incubated with Flag‐APRIL first, and plasma was added afterwards. X‐axis shows fluorescence on a log scale (10^2^–2 × 10^5^). The experiment was performed three times. (d) Triglyceride lipase activity was measured in plasma of mice (*n* = 13) before or after heparin administration, using a quenched fluorescent triglyceride (TG) derivative as substrate. The assay was performed twice in the presence of an excess of heparin. *P<.05, significantly different as indicated; paired *t‐*test. (e) A standard curve of active atacicept was measured by its ability to protect BAFFR:Fas reporter cells from Fc‐BAFF‐induced death. The experiment was performed three times. (f) Active atacicept was measured in mouse plasma before (*n* = 5), or 1 day (*n* = 5), 3 days (*n* = 5), or 7 days (*n* = 1) after atacicept administration. Active atacicept concentrations were derived from the EC_50_ of Fc‐BAFF inhibition. Values obtained in plasma of heparin‐treated mice were corrected by the blood dilution factor caused by heparin administration. The dotted line shows the lower limit of quantification. Measures were performed in duplicate. The experiment was done three times with identical results. (g) A standard curve of atacicept diluted in mouse plasma was measured by AlphaLISA, in the presence or absence of 200 ng·L^−1^ of recombinant mouse BAFF, using a matched pair of anti‐TACI antibodies. (h) Same as panel (g), except that samples were submitted to an acid dissociation procedure to separate BAFF from atacicept. Experiments of panels (g) and (h) were performed five times. (i) Mice received atacicept (or not, *n* = 6) subcutaneously at Day 0. Plasma was taken at Days 1 (*n* = 6), 3 (*n* = 5, because not enough plasma was left from the sixth mouse), 7 (*n* = 6), or 14 (*n* = 10), before (circles) and after (triangles) intravenous administration of heparin. Atacicept was measured by AlphaLisa in plasma samples without (white symbols) or with (black symbols) an acid dissociation step. Values of heparin‐treated mice were corrected by the dilution factor induced by heparin administration. Measures were performed four times on the same set of samples, with similar results. *P<.05, significantly different as indicated; one‐way ANOVA was performed three times for conditions at Days 3, 7, and 14, respectively, with Bonferroni's multiple comparison tests at Days 7 and 14. Statistical analysis was performed on log‐transformed data; because under these conditions, there were no significant variance inhomogeneity. (j) A standard curve of mouse BAFF diluted in serum of a *Baff* ^*−/−*^ mouse was measured by AlphaLISA, in the presence of 0, 100, or 2000 ng·ml^−1^ of atacicept, using a matched pair of anti‐mBAFF antibodies. The experiment was performed three times. (k) Circulating BAFF levels in plasma of WT mice treated subcutaneously at Day 0 with a single dose of atacicept. Plasma was collected at Day 0 (before atacicept administration), Day 1, Day 3, Day 7, or Day 14 after atacicept administration, and before or after administration of heparin on the day of plasma collection. BAFF levels were calculated using the standard curve of BAFF in the absence of atacicept and are expressed as “unbound mBAFF equivalent.” Samples were measured three times independently, with similar results. APRIL, a proliferation‐inducing ligand; BAFF, B cell activating factor of the TNF family; BAFFR, BAFF receptor; TACI, transmembrane activator and calcium‐modulator and cyclophilin ligand (CAML)‐interactor

HEK293 cells are only partly representative of the cellular diversity present in a living organism, and it was therefore still possible that atacicept, or putative atacicept multimers that would be formed post‐administration, would interact with proteoglycans in vivo. Thus, mice were treated first with atacicept and then rested for 1, 3, 7, or 14 days. At these time points, plasma was collected before or 20 min after intravenous administration of a high dose (1,000 IU·kg^−1^) of heparin. Heparin activity in plasma of treated mice was high in all cases (6–22 IU·ml^−1^, on average 15 IU·ml^−1^), indicating successful administration (Figure 6a). Several lines of evidence indicate that heparin should have been able to detach proteoglycan‐bound molecules in the vasculature: First, in 293T cells, an incubation as short as 5 min with 0.1 IU·ml^−1^ of heparin was sufficient to fully detach pre‐bound Flag‐APRIL (Figure 6b). Second, heparin in plasma post‐treatment was sufficient to compete with Flag‐APRIL binding to 293T cells (Figure 6c). Third, heparin treatment significantly increased triglyceride lipase activity in plasma, suggesting that it had mobilized endogenous proteoglycan‐bound lipoprotein lipase (Figure 6d). Levels of active atacicept were determined in plasma, before and after heparin administration, using a reporter cell assay that can measure active atacicept across a wide range of concentrations (Figure 6e). No change in active atacicept was detected before versus after heparin administration (Figure 6f). This, however, did not exclude that heparin could have released a ligand‐bound version of atacicept. This ligand‐occupied atacicept would not be detected in the active atacicept assay. Thus, AlphaLisa was used to measure total levels of atacicept. As this assay detects ligand‐bound atacicept less efficiently than unbound atacicept (Figure 6g), the assay was also performed after an acid dissociation step that overcomes ligand‐induced quenching of the detection signal (Figure 6h). In the absence of acid dissociation, average atacicept levels peaked at 9 μg·ml^−1^ at Day 1 and then decreased to 1.5, 0.6, and 0.3 μg·ml^−1^ after Days 3, 7, and 14. After acid dissociation, average measured values for atacicept were 8, 1.6, 1.2, and 0.9 μg·ml^−1^ at Days 1, 3, 7, and 14, respectively (Figure 6i). Significantly higher atacicept levels detected after acid dissociation indicate the accumulation of ligand‐bound atacicept at late time points (Figure 6i), a conclusion reinforced by the measure of BAFF in plasma that increased to high levels at Days 7 and 14, despite the fact that BAFF detection was attenuated by the presence of atacicept in this experimental readout (Figure 6j,k). No differences were observed for levels of active or total atacicept before versus after high dose heparin administration, arguing against the hypothesis that a significant pool of proteoglycan‐bound atacicept, free or ligand‐bound, exists in the vasculature (Figure 6f,i).

## 4 DISCUSSION

The structure of heparin, a repeated disaccharide unit of variably sulfated iduronic acid (mainly 2‐O‐sulfated) and *N*‐acetyl glucosamine (mainly 6‐O‐ and N‐sulfated), is very similar to that of glycosaminoglycan side chains of heparan sulfate proteoglycans and proteins that bind these glycosaminoglycans side chains also bind to heparin (Xu & Esko, 2014). As the BAFF and APRIL receptor TACI interacts with syndecan‐2 and other proteoglycans (Bischof et al., 2006), it was necessary to address the question of a potential drug interaction of heparin and atacicept, which contains a part of the extracellular domain of TACI. No binding of atacicept to cell surface proteoglycans was detected. An attempt to map the proteoglycan‐binding site on TACI first yielded intriguing results: Purified dimeric TACI‐Fc did not bind detectably to proteoglycans, whatever the TACI sequence contained in these constructs. Weak but specific binding was only seen with some TACI‐Fc preparations that had not been size‐fractionated, or with high MW fractions that usually also contained various contaminating proteins. However, after treatment with cysteine, two or more atacicept dimers were cross‐linked via disulfide bridges. These atacicept oligomers displayed robust binding to proteoglycans, suggesting that binding to proteoglycans is an intrinsic, avidity‐dependent TACI property, and not the result of an indirect binding via contaminants. Oligomerization of atacicept through binding to BAFF 60‐mer also increased binding to proteoglycans. With this later assay, all TACI constructs containing either CRD1 and/or CRD2 interacted with proteoglycans, as well as the few BCMA and BAFFR constructs that were tested, suggesting that weak proteoglycan binding might be a general property of BAFF‐binding CRDs, and/or of the Fc portion, and not of a putative distinct cluster of positively‐charged amino acid residues. Our results showing that atacicept and other dimeric TACI‐Fc constructs do not bind to proteoglycans do not rule out that TACI may physiologically interact with proteoglycans, possibly after self‐ or ligand‐induced multimerization. Discrepant or inconsistent results obtained across studies for binding of TACI‐Fc to proteoglycans could simply reflect varying amounts of aggregates in TACI‐Fc preparations that had not always been size‐fractionated.

As dimeric atacicept does not bind to proteoglycans, it was expected not to interfere with the activity of heparin, and it did not. Atacicept in complex with APRIL did not affect heparin activity up to an atacicept concentration of 100 μg·ml^−1^, which is at least 10‐fold higher than could be achieved with atacicept in vivo. Even artificially multimerized atacicept, which binds to proteoglycans, did not change heparin activity. The lack of interference of high MW atacicept or atacicept–APRIL complexes with heparin activity can be explained by the observation that the affinity of heparin for different proteins can differ by up to three or four orders of magnitude (Xu & Esko, 2014). The affinity of APRIL for proteoglycans in BCMA‐ and TACI‐negative cell lines was measured in the range of 20–80 μM (Dillon, Gross, Ansell, & Novak, 2006), whereas the affinity of heparin for anti‐thrombin is 3–20 nM, that is, 1000‐fold stronger (Bjork et al., 1992; Xu & Esko, 2014).

Heparin binds APRIL and might indirectly alter its binding to atacicept. The binding site of TACI to APRIL has been characterized in detail in a co‐crystal structure (Hymowitz et al., 2005), showing that the interaction takes place on a face of APRIL that is opposite to the basic stretch known to facilitate heparin binding (Hendriks et al., 2005; Ingold et al., 2005). This does not support the hypothesis that heparin could interfere with the APRIL–TACI interaction, and our results indeed showed no interference of heparin on the ability of atacicept to inhibit BAFF or APRIL, even at heparin concentrations higher than those achieved in clinical practice. Doses of heparin used in the clinic vary with the desired anti‐coagulant action. For example, after a heart attack or a thrombosis, doses of up to 10 000 IU heparin can be given intravenously (Unknown, 2011), which would result in plasma concentrations of about 2 IU·ml^−1^. Heparin up to concentrations of 32 IU·ml^−1^ did not affect atacicept, but decreased activity of recombinant Fc‐APRIL about twofold in a reporter cell assay. We speculate that the docking of Fc‐APRIL on cell surface proteoglycans increases its local concentration and agonistic activity on reporter cells and that this effect is reduced in the presence of heparin that prevents binding of Fc‐APRIL to the surface of the reporter cells. Heparin was shown previously to inhibit the growth factor activity of APRIL on multiple myeloma cells by preventing its binding to proteoglycans (Moreaux et al., 2009). We have further observed that TACI (and possibly BCMA) stimulation requires ligand oligomerization (Bossen et al., 2008). In line with this result, APRIL activity on primary B cells is enhanced by proteoglycans or heparan sulfate proteoglycans that would cross‐link and position the TACI/BCMA binding domain of APRIL, making it a better activator of receptors (Kimberley et al., 2009). Thus, although heparin is unlikely to interact with atacicept, it might interfere with the biology of APRIL–proteoglycan interactions, with a potential modulation of plasma cell biology in a physiological setting. It will be interesting in the future to study the effects of heparin on the biology of endogenous APRIL, although this might be complicated by the vast array of proteins whose interactions with proteoglycans will also be modulated by heparin (Xu & Esko, 2014).

In conclusion, our results provide no indication that atacicept interferes with heparin and vice versa, providing no reason to expect atacicept–heparin drug interactions.

## AUTHOR CONTRIBUTIONS

D.W., E.S., Ö.Y., and P.S. designed the study. C.K.Q., L.W., S.S.M., B.P., M.E., M.V., and P.S. performed experiments and acquired data. D.W., M.G and Ö.Y. contributed essential reagents. H.B. made key intellectual contributions. P.S. wrote the article. All the authors critically revised the manuscript for important intellectual content and approved the submitted version.

## CONFLICT OF INTEREST

P.S. is supported by a research grant from Merck KGaA. D.W., M.G, H.B., and Ö.Y. are employees of Merck KGaA. E.S. is an employee of EMD Serono. Other authors declare no conflicts of interest.

## DECLARATION OF TRANSPARENCY AND SCIENTIFIC RIGOUR

This Declaration acknowledges that this paper adheres to the principles for transparent reporting and scientific rigour of preclinical research as stated in the *BJP* guidelines for https://bpspubs.onlinelibrary.wiley.com/doi/full/10.1111/bph.14207, https://bpspubs.onlinelibrary.wiley.com/doi/full/10.1111/bph.14208, and https://bpspubs.onlinelibrary.wiley.com/doi/full/10.1111/bph.14206, and as recommended by funding agencies, publishers and other organisations engaged with supporting research.

## Data Availability

The authors declare that the data supporting the findings of this study are available within the article, in its supplementary information files and as a dataset doi: https://doi.org/10.5281/zenodo.3350386.
